# Glacial History Affected Phenotypic Differentiation in the Alpine Plant, *Campanula thyrsoides*

**DOI:** 10.1371/journal.pone.0073854

**Published:** 2013-10-16

**Authors:** J. F. Scheepens, Eva S. Frei, Jürg Stöcklin

**Affiliations:** Section of Plant Ecology, Institute of Botany, University of Basel, Basel, Switzerland; University of Gottingen, Germany

## Abstract

Numerous widespread Alpine plant species show molecular differentiation among populations from distinct regions. This has been explained as the result of genetic drift during glacial survival in isolated refugia along the border of the European Alps. Since genetic drift may affect molecular markers and phenotypic traits alike, we asked whether phenotypic differentiation mirrors molecular patterns among Alpine plant populations from different regions. Phenotypic traits can be under selection, so we additionally investigated whether part of the phenotypic differentiation can be explained by past selection and/or current adaptation. Using the monocarpic *Campanula thyrsoides* as our study species, a common garden experiment with plants from 21 populations from four phylogeographic groups located in regions across the Alps and the Jura Mountains was performed to test for differentiation in morphological and phenological traits. Past selection was investigated by comparing phenotypic differentiation among and within regions with molecular differentiation among and within regions. The common garden results indicated regional differentiation among populations for all investigated phenotypic traits, particularly in phenology. Delayed flowering in plants from the South-eastern Alps suggested adaptation to long sub-mediterranean summers and contrasted with earlier flowering of plants experiencing shorter growing seasons in regions with higher elevation to the West. Comparisons between molecular and phenotypic differentiation revealed diversifying selection among regions in height and biomass, which is consistent with adaptation to environmental conditions in glacial refugia. Within regions, past selection acted against strong diversification for most phenotypic traits, causing restricted postglacial adaptation. Evidence consistent with post-glacial adaptation was also given by negative correlation coefficients between several phenotypic traits and elevation of the population's origin. In conclusion, our study suggests that, irrespective of adaptation of plants to their current environment, glacial history can have a strong and long-lasting influence on the phenotypic evolution of Alpine plants.

## Introduction

Glacial history, containing recurring processes of retreat, glacial survival and recolonisation of species [1], has had major consequences for intraspecific evolution of widespread Alpine plants [2],[3] and has likely led to numerous allopatric speciation events [1], [4]. The long-term glacial survival of Alpine plant species in isolated refugia in the periphery of the European Alps likely caused genetic drift, which explains the strong spatial genetic structure demonstrated in various molecular studies (*e.g.* [2], [5], [6]).

It can be hypothesized that in widespread Alpine plants, glacial survival not only caused differentiation of neutral molecular markers due to genetic drift, but also of phenotypic traits in a similar way through processes of genetic drift, natural selection or a combination of both [1], [7], , for two important reasons: the time scale of glacial survival is long enough for genetic drift and mutations to arise, leading to phenotypic differentiation of isolated refugial populations [2], [9]; besides these neutral processes, long-term selection in isolated refugia may have caused adaptation to environmental conditions in refugia. Similarly, differentiation in phenotypic traits among populations of phylogeographic groups can be expected to result from processes acting during the more recent post-glacial recolonisation: founder events and bottlenecks are likely during rapid postglacial recolonisation of the Alps, causing phenotypic differentiation of former refugial populations and their derived populations [4]; additionally, adaptations to current local environmental conditions in the heterogeneous Alpine landscape, especially to factors correlating with elevation [7], [10], may have appeared during and after post-glacial recolonisation.

Our study species, the monocarpic perennial *Campanula thyrsoides* L., is genetically subdivided into four major phylogeographic groups located in regions arranged longitudinally across the European Alps (henceforth *phylogeographic regions*; Fig. S1 in Supporting Information; [11]–[13]) as has been found in other Alpine plant species (*e.g.* [2], [5]). The regional genetic arrangement is congruent with major biogeographic distribution patterns based on floristic data [14], [15] and the coincidence of allelic and species break zones has recently established this link [3].

We hypothesized that the phylogeographic pattern of neutral molecular marker differentiation in *C. thyrsoides*, being a result of long-term glacial survival in refugia, is mirrored in phenotypic trait differentiation, independent of whether this is caused by genetic drift, past selection processes or both. We tested this hypothesis by measuring phenotypic traits of plants of 21 populations sampled from across the species' distribution and grown in a common garden, allowing for the quantification of genetic trait differentiation among regions, populations and seed families. *Campanula thyrsoides* frequently occurs in sub-alpine/alpine pastures and meadows [16], and we therefore included a clipping treatment to simulate grazing, or mowing, in order to investigate plant responses to this stressor. The clipping treatment also served as experimental insurance: all untreated plants may perform similar to each other due to benign conditions in the common garden, and differentiation may become apparent only in the treated, *i.e.* more stressed, plants.

Reciprocal transplantation experiments would be needed to prove local or regional adaptation [17], but results from other methods may give evidence consistent with adaptation, such as *Q*_ST_-*F*_ST_ analysis [18], [19] and correlations between traits and local environmental conditions [20], [21]. We compared quantitative trait differentiation among and within phylogeographic regions and compared molecular with quantitative differentiation to infer past selection among and within regions, presuming that among-region differentiation is shaped during glacial survival in peripheral refugia whereas within-region differentiation is formed after the start of post-glacial recolonisation (*cf.* [22], [23]). We also investigated post-glacial adaptation by calculating correlation coefficients between phenotypic traits and the elevation of origin across the sampled populations.

## Materials and Methods

### Ethics Statement

Our study species is not officially endangered, red-listed or otherwise protected at state level in the countries where the sampling was conducted. We did not need specific permission to take seed samples from the publicly accessible natural populations. We had an official agreement with the owners of the common garden site that allowed us to perform our study on their property.

### Study species

**Figure 1:**
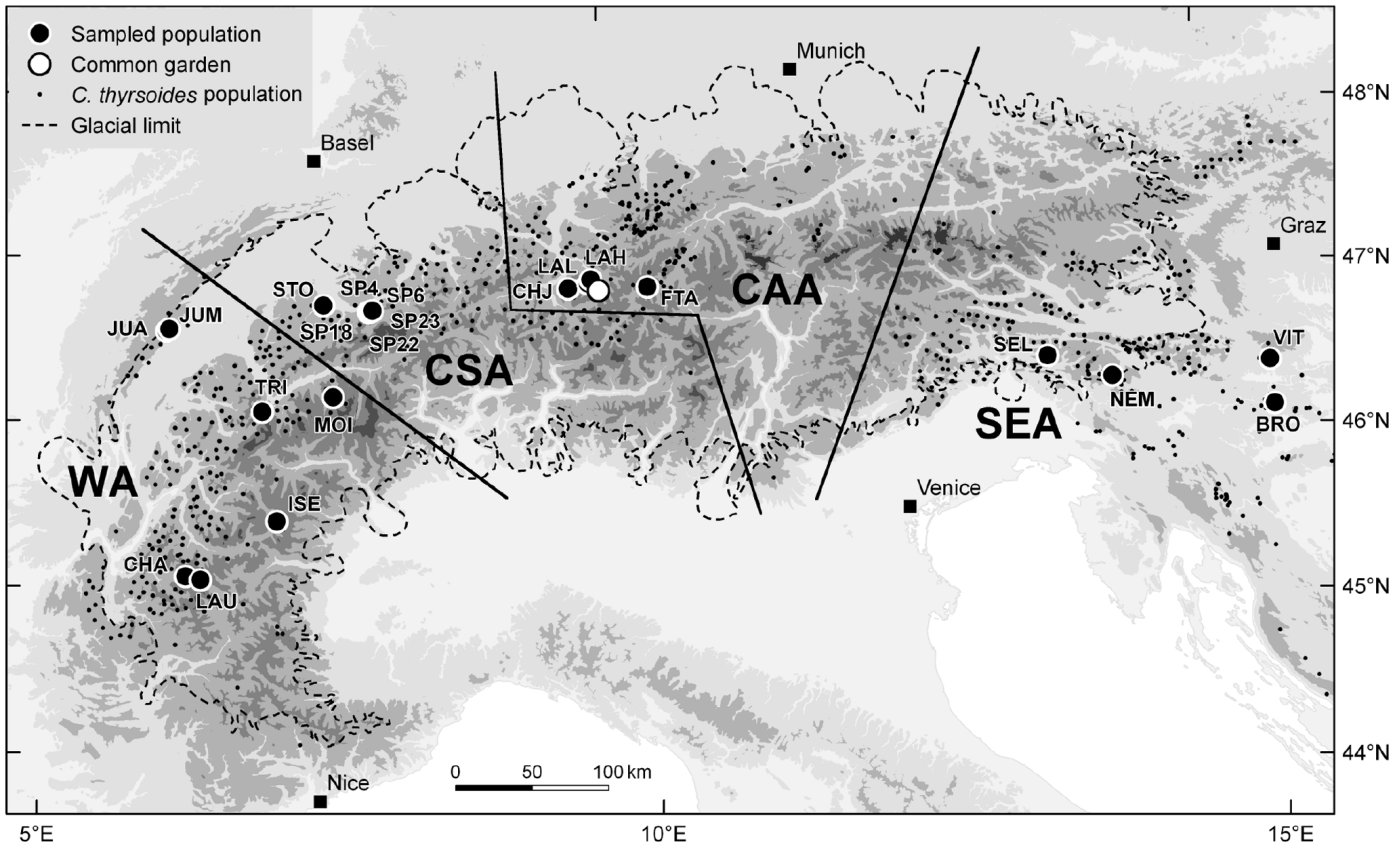
Map of the European Alps showing the 21 sampled *Campanula thyrsoides* populations. Populations are divided into four phylogeographic regions: WA – Western Alps; CSA – Central Swiss Alps; CAA – Central Austrian Alps; SEA – South-eastern Alps. Lines delineating the regions are schematic [12]. Population abbreviations are spelled out in Table S1. Data on the distribution of *Campanula thyrsoides* were kindly provided by Dr. Erik Welk, Department of Geobotany, Martin Luther University of Halle-Wittenberg. Data on the glacial limit from the Last Glacial Maximum were obtained from Kuss et al. [2]. Map projection: Mollweide.

*Campanula thyrsoides* (Campanulaceae) was used for this study because of its Alpine-wide distribution [16]. This monocarpic bell flower occurs in grasslands on calcareous soils or carbonate-rich schists, frequently in systems moderately disturbed by natural (steep slopes with unstable soil) and/or managed causes (mowing or grazing; [11]). Four phylogeographic regions of *C. thyrsoides* (Fig. 1), delimited based on structure in nuclear microsatellite data revealed by Bayesian clustering analysis (Fig. S1; [11]), are roughly located (i) in the Jura Mountains and Western Alps (WA) from Nice to Aosta, (ii) in the Central Swiss Alps (CSA) from Aosta to Lake Como, (iii) in the Central Austrian Alps (CAA) from Lake Como to the Dolomites and (iv) in the South-eastern Alps (SEA) from the Dolomites eastwards. Two morphologically, geographically and ecologically distinct subspecies have been recognised: subsp. *thyrsoides* in the Jura Mountains and most of the European Alps (phylogeographic regions WA, CSA, CAA and less frequently in SEA), and subsp. *carniolica* in the South-eastern Alps (SEA) [24] and the Dinarids. The elevational distribution of subsp. *thyrsoides* typically ranges from 1,600 to 2,200 m a.s.l. [11], but reaches lower elevations in the Jura Mountains (ca. 1400 m a.s.l.) and the South-eastern Alps (down to 1200 m a.s.l.). Subsp. *carniolica* has a lower elevational distribution in the South-eastern Alps (ca. 1000 m a.s.l.) and the Dinarids (ca. 400 m a.s.l.), with the lowest population recorded at 217 m a.s.l. near Gračnica, Slovenia (Jürg Stöcklin, pers. obs.). The molecular differentiation between the two subspecies (AMOVA; 8.4%) is higher than that between the phylogeographic regions WA and CSA+CAA within subspecies *thyrsoides* (6.0%), whereas regions CSA and CAA are only slightly differentiated (2.3%), potentially due to postglacial admixing between these regions [12]. The two subspecies are able to produce viable seeds from artificial crosses (J.F. Scheepens, H. Kesselring, J. Stöcklin, unpublished data).

*Campanula thyrsoides* is characterised by isolated populations of several hundred to a few thousand individuals [25]. Initiation of flowering depends on rosette size, and the average flowering age was estimated to be about 10 years using integral projection models and herb chronology [25]. However, flowering age is highly variable (3–16 years; [11]), and under benign conditions in a common garden, the majority of plants flowers in the second year [26]. This out-crossing species has a strong but incomplete self-incompatibility system [27].

### Common garden experiment

Six seed families from each of 21 populations were sampled in the four phylogeographic regions across the Alps and the Jura Mountains (Fig. 1, Table S1 in Supporting Information). This design was optimal for subsequent *Q*_ST_ analysis given the available resources [28]. Because we selected populations before phylogeographic analysis was completed, we could not foresee that three of four populations from CAA showed strong admixture with CSA (Fig. S1). Nevertheless, we treated these three populations as belonging to CAA as a separate region. From September 2007, randomly chosen seeds were germinated on moist filter paper in Petri dishes in a greenhouse located in Basel, Switzerland (276 m a.s.l.). Eight seedlings per seed family were planted into pots of 4 cm diameter filled with low-nutrient soil (Anzuchterde, Ökohum, Herrenhof, Switzerland). Plants were repotted after 10–18 weeks into pots of 10×10×10 cm with potting soil (Topferde, Ökohum, Herrenhof, Switzerland). Fertiliser (Wuxal, Maag, Düsseldorf, Germany) was added once. In spring, plants were transferred outside the greenhouse to acclimatise before final transplantation.

On 19 May 2008, plants were transplanted to a common garden located at 1,530 m a.s.l. in Davos, Graubünden, Switzerland (N 46°47′06.97”, E 9°48′57.02”; Fig. 1). We chose this location to let plants experience a sub-alpine climate, to which the plants' life cycle is presumed to be relatively well adapted. The privately owned site, formerly used as an organically fertilised sub-alpine meadow-pasture, was ploughed before plants were transplanted into the local soil. Out of 1008 plants, 953 individuals could be transplanted (Table S1), whereas the remaining 55 could not, due to mortality in the greenhouse. Rainfall at the common garden location averages 1,026 mm per year and minimum, mean and maximum temperature averages are −8.2°C, 2.9°C and 15.1°C respectively (WorldClim data; [29]). The experimental site was fenced and plant beds were regularly weeded.

During transplantation, rosette diameter was measured. Eight weeks after transplantation, on 15 July 2008, a clipping treatment to simulate herbivory was applied to half of the plants of each seed family. Using scissors, we cut off all leaves as close as possible to the rosette centre without injuring the apical meristem. Around the end of the growing season, on 9 September 2008, leaf length and width of the longest leaf and number of leaves were measured. Since oblongate spring leaves are replaced in summer for obovate leaves in this species [30], leaf length and width of the longest leaf were measured again on 1 June 2009. The leaf length to width ratio was calculated using both 2008 and 2009 data separately. For each flowering plant, the number of inflorescences, the height and the number of flowers were measured on 27 July 2009, and again on 20 October 2009 for most SEA plants; the last measured values were used for analyses. The above-ground biomass was harvested when plants finished flowering and was weighed after drying for 72 hours at 60°C in an oven. During each visit, phenological states were recorded, using the classes *dead*, *rosette*, *bolting* (*i.e.* initiation of flowering), *flowering* (*i.e.* at least one flower in anthesis) and *ripening* (*i.e.* when all flowers were wilted). From this data, post-transplantation survival (*i.e.* still alive on 15 July 2008) was deduced.

### Generalized linear mixed-effect models

Data on post-transplantation survival, leaf length to width ratio in 2008 and 2009, number of leaves, number of inflorescences, maximum inflorescence height, number of flowers and above-ground biomass were analysed using generalized linear mixed-effect models (GLMMs) [31]. Analyses were performed using the R statistical package [32] (version 2.10.1) with the command *lmer* from package “lme4” [33]. We applied Type I sums of squares, which allowed for first removing the covariate effects, then testing the treatment factor, thus leaving the residual variation to be explained by the origin effects of region, population and seed family. We checked robustness of results by comparing models with different factor sequences as long as the order made analytical sense [31].

In all models, the rosette diameter at the start of the experiment was included as a covariate to account for effects of initial size on the measured variables. Next, we included the mean Euclidean geographic distance of each focal population to all other populations as a covariate to account for geographic distance-related effects of genetic drift or large-scale environmental gradients, which on their own may cause a pattern of seemingly regional differentiation. The clipping treatment was included as a fixed effect in all models, except in the model testing post-transplantation survival, as this trait was assessed before the clipping treatment was applied. Phylogeographic region (fixed), population (random) and seed family (random) were nested, and so were interactions of the clipping treatment with region (fixed), population (random) and seed family (random).

Survival after transplantation was analysed using a binomial error distribution with a logit-link function, and number of inflorescences was analysed using a quasi-Poisson error distribution with a log-link function. Number of leaves and number of flowers fit a normal distribution better than a Poisson distribution, so these and the remaining response variables were analysed with a normal error distribution. For all traits analysed with normal error expectations, the normality of full model residuals and homogeneity of variances were checked visually by constructing diagnostic plots. To improve normality of the model residuals, we used power transformations [31]: [number of leaves]^0.656^, [number of flowers]^0.620^ and [above-ground biomass]^0.331^. The left-skewed model residuals of maximum inflorescence height violated normality considerably, and transformations only worsened normality, but since non-normality was not due to outliers, untransformed data were used.

To test the significance of model factors, we calculated χ^2^-values and *P*-values from likelihood ratio tests of model comparisons using maximum likelihood, starting with the deletion of the interactions and removing factors subsequently until all factors had been tested. Variance component analyses were performed on the full models treating all factors as random and using restricted maximum likelihood [31]. Tukey's HSD tests were used to assess differences between region pairs. Finally, *G* tests [34] were performed on data on phenological states to test for differences among regions and populations within regions.

### Past selection

To infer past selection, we calculated phenotypic and molecular differentiation indices among and within phylogeographic regions. To this end, we performed linear random effect models including region, population and seed family as well as the covariate, rosette diameter at the start of the experiment, using trait response variables from unclipped plants only. We then used the resulting variance components to calculate the indices, *Q*_RT_ and *Q*_SR_ [18], . These represent trait differentiation among regions and among populations within regions, respectively [23], and are assumed to reflect differentiation during glacial survival and after postglacial recolonisation, respectively. Previously obtained molecular data from five microsatellite loci from the sampled populations [12], [13] were used to calculate with GenAlEx [35] the standardised neutral molecular differentiation indices, *F*'_RT_ and *F*'_SR_. We refer to Ægisdóttir et al. [12] and Kuss et al. [13] for details about the molecular analysis. The differentiation index *F*' is based on standardised AMOVAs [36] and is an improved version of Hedrick's [36] corrected index for molecular differentiation, *G*'. Instead of the Schynige Platte populations SP4, SP6, SP18, SP22 and SP23, which were not genotyped, we used genetic data from two other populations nearby as a replacement (SPO and SPU in Ægisdóttir et al. [13]). The 95% confidence intervals of the *Q* and *F*' indices were generated by jackknifing over populations, which performs reasonably well compared to parametric bootstrapping [37]. Molecular and phenotypic differentiation indices were then compared to infer past selection. If *Q* > *F*, trait differentiation is larger than can be expected based on neutral differentiation, thus indicating diversifying selection. The opposite, *Q* < *F*, indicates unifying selection. Selection need not be invoked to explain *Q*  =  *F*, as this can be the result of genetic drift alone.

### Local adaptation

To explore current adaptation, Pearson's correlations were performed between population-averaged trait values of unclipped plants, which were calculated from seed family means, and elevation of population origin (Table S1), which was used as a proxy for environmental variables related to elevation. Elevation of population origin correlates (*r* = 0.68, *P*<0.001) with the first principal component of climatic data (precipitation and minimum, mean and maximum temperature; WorldClim data [29]; data not shown), which in turn explains 99.97% of the variation in these climatic variables. The climatic differences between the sub-alpine/alpine regions (WA, CSA, CAA) versus the sub-mediterranean region (SEA) could disrupt gradients in phenotypic traits [26]. To account for this, we performed correlations with a subset containing WA, CSA and CAA populations (subsp. *thyrsoides*) as well as with a subset containing SEA populations (subsp. *Carniolica*).

## Results

### Phenotypic differentiation

**Figure 2:**
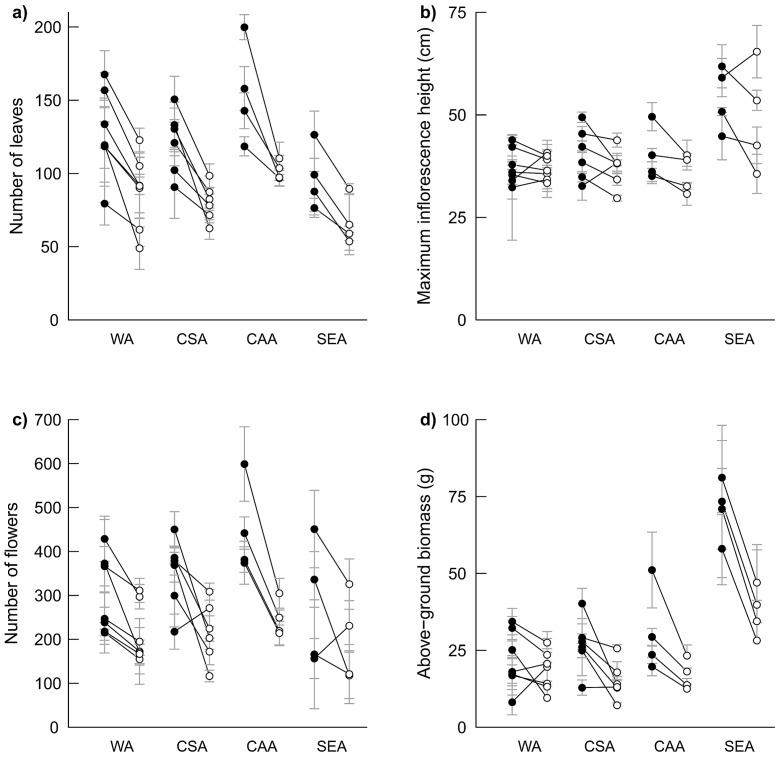
Response to clipping of *Campanula thyrsoides* plants from 21 populations grown in a common garden. Populations are ordered by phylogeographic region for (a) number of leaves, (b) maximum inflorescence height, (c) number of flowers, and (d) above-ground biomass. Filled circles indicate means of control plants, open circles indicate means of clipped plants. Error bars (grey) indicate standard errors (±1 SE) based on family means. WA – Western Alps; CSA – Central Swiss Alps; CAA – Central Austrian Alps; SEA – South-eastern Alps.

Regional differentiation was significant in all traits (Table 1, Fig. 2). Tukey's HSD tests indicated that post-transplantation survival was higher in CSA populations compared to WA populations, number of leaves was higher in CAA than in SEA plants, maximum inflorescence height was higher in SEA than in WA plants, and SEA populations showed higher above-ground biomass compared to populations from the other regions (Table 2, Fig. 2). No significant differences between pairs of regions could be found for leaf length to width ratios, number of inflorescences and number of flowers (Table 2), which can be attributed to low statistical power. Populations within regions were significantly different for all traits except survival, and seed families within populations were significant for all traits except survival and number of flowers (Table 1).

**Table 1:** Results of generalised linear mixed-effect model analysis on eight phenotypic traits of *Campanula thyrsoides* measured in the common garden.

		Post-transplantation survival			Leaf length to width ratio 2008			Leaf length to width ratio 2009			Number of leaves			Number of inflorescences			Maximum inflorescence height			Number of flowers			Above-ground biomass		
	df	χ^2^		*%VC*	χ^2^		*%VC*	χ^2^		*%VC*	χ^2^		*%VC*	χ^2^		*%VC*	χ^2^		*%VC*	χ^2^		*%VC*	χ^2^		*%VC*
Initial rosette diameter^1^	1	479.0	***	62.8	94.3	***	4.0	52.1	***	3.3	156.7	***	16.3	15.4	***	0.1	52.5	***	0.0	34.9	***	0.3	85.1	***	10.4
Geographic distance^1^	1	0.0		2.6	14.7	***	8.9	0.3		3.7	43.4	***	3.6	41.2	***	1.9	117.7	***	6.6	0.8		4.0	51.4	***	1.9
Clipping treatment^2^	1	-		-	1.2		0.2	36.7	***	6.4	155.9	***	23.5	49.8	***	1.6	19.5	***	2.7	95.5	***	21.5	77.8	***	14.8
Region^2^	3	24.0	***	18.3	35.0	***	9.0	19.9	***	0.0	17.1	***	2.6	37.3	***	1.0	67.5	***	23.9	43.9	***	0.8	39.8	***	15.9
Population (Region)^3^	1	0.4		2.6	108.9	***	8.9	46.8	***	3.7	68.8	***	3.6	38.7	***	1.6	39.0	***	6.5	18.6	***	5.9	10.6	**	1.9
Seed family (Population)^3^	1	0.6		0.5	19.4	***	8.4	9.2	**	7.9	4.1	*	2.2	16.0	***	2.3	17.9	***	9.7	0.0		1.6	4.6	*	4.7
Clipping×Region^2^	3	-		-	2.4		0.0	2.2		0.0	4.4		0.0	1.8		0.0	7.6	(*)	0.0	7.7	(*)	1.0	18.7	***	3.0
Clipping×Pop (Region)^3^	1	-		-	0.1		0.8	4.4	*	5.5	0.1		0.4	0.0		0.0	7.1	**	5.3	0.0		1.9	1.1		1.8
Clipping×Seed family (Population)^3^	1	-		-	0.0		0.0	0.0		0.0	0.5		2.8	0.0		0.0	0.0		0.0	0.0		0.0	0.0		0.0
Residuals	609– 942			13.1			60.0			69.4			44.9			91.5			45.4			62.9			45.5

1 Covariate; ^2^ Fixed effect; ^3^ Random effect. χ^2^ values and their significances were obtained from model comparisons. *%VC* – Variance components were obtained from analyses with all factors treated as random effects. df – degrees of freedom, residual df varies per trait due to mortality and due to flowering traits being recorded in flowering plants only. (*) *P* = 0.054; * *P*<0.05; ** *P*<0.01; *** *P*<0.001.

**Table 2:** Mean values of morphological traits of *Campanula thyrsoides* per phylogeographic region and treatment (control versus clipped plants) measured in the common garden.

	Region				Treatment	
	WA	CSA	CAA	SEA	Control	Clipped
Post-transplantation survival (%)	76.3^a^ (10.2)	94.5^b^ (3.1)	99.0^ab^ (0.6)	78.7^ab^ (7.4)	-	-
Leaf length to width ratio 2008	2.71 (0.11)	2.62 (0.09)	3.27 (0.24)	3.18 (0.33)	2.92 (0.15)	2.99 (0.16)
Leaf length to width ratio 2009	7.25 (0.42)	6.86 (0.36)	7.26 (0.45)	7.18 (0.57)	7.70^a^ (0.10)	6.77^b^ (0.24)
Number of leaves	106.3^ab^ (9.7)	100.0^ab^ (6.7)	128.3^a^ (9.9)	82.4^b^ (9.1)	126.0^a^ (11.7)	84.1^b^ (7.4)
Number of inflorescences	4.06 (0.55)	4.86 (0.23)	4.65 (0.31)	6.22 (0.87)	5.6^a^ (0.4)	4.3^b^ (0.5)
Maximum inflorescence height (cm)	37.21^a^ (1.26)	38.86^ab^ (2.21)	37.58^ab^ (2.50)	52.33^b^ (4.62)	43.1^a^ (3.8)	39.8^b^ (3.2)
Number of flowers	254.7 (31.0)	285.5 (23.3)	335.3 (33.7)	241.1 (66.4)	347.8^a^ (38.3)	217.9^b^ (10.3)
Above-ground biomass (g)	18.89^a^ (2.67)	20.96^a^ (2.62)	23.15^a^ (4.52)	53.40^b^ (4.44)	38.1^a^ (11.1)	21.9^b^ (5.2)

Means (SE) of regions are based on population means, which in turn are based on seed family means. Different superscript letters indicate significant differences (α = 5%) among regions using Tukey's HSD tests and between treatments based on significance of the treatment factor in the generalized linear mixed-effect models (Table 1). WA – Western Alps; CSA – Central Swiss Alps; CAA – Central Austrian Alps; SEA – South-eastern Alps.

The mean Euclidean geographic distance of each focal population to all other populations, used to account for any confounding effect of seemingly regional differentiation, was significant for leaf length to width ratio in 2008, number of leaves, number of inflorescences, maximum inflorescence height and above-ground biomass (Table 1), albeit with moderate amounts of variance explained (1.9–8.9%). However, excluding this factor from the analysis did not increase variance explained at the region level and only slightly increased variance explained at the population level (data not shown).

The clipping treatment significantly reduced all trait values except for leaf length to width ratio in 2008 (Tables 1 & 2, Fig. 2). A clipping×region interaction was found for above-ground biomass (Table 1), with WA plants showing on average less strong responses to clipping compared to plants from other regions (Fig. 2). There were significant clipping × population interactions for leaf length to width ratio in 2009 and maximum inflorescence height (Table 1), which indicates genetic variability in strength and direction of the response to clipping among populations. Several populations responded positively to clipping in maximum inflorescence height, number of flowers and above-ground biomass (Fig. 2). Clipping × seed family interactions were never significant, indicating that all seed families within a population responded similarly to the clipping treatment.

Rosette diameter at the start of the experiment affected the outcome of all dependent variables significantly (Table 1), but the amount of variance explained by this factor was highly variable among traits. It strongly and positively affected survival after transplantation (63%), and had a substantial effect on number of leaves (16%) and biomass (10%). Variation in height, number of inflorescences and flowers explained by initial rosette diameter was negligible (0.0–0.3%).

The models explained the data to variable degrees, ranging from only 8.5% of variance explained in number of inflorescences to 86.9% in post-transplantation survival (Table 1). Due to considerable orthogonality in the design, changing the position of explanatory factors (while respecting the nesting structure) had only negligible influence on the results (results not shown).

Out of the total 953 plants, 132 individuals died between transplantation and the second measurement. This post-transplantation survival was significantly dependent on phylogeographic region, which explained 18% of variation, as WA and SEA plants had lower survival compared to plants from the two central regions (Tables 1 & 2). Only fifteen plants died between the second and third measurement in the first season, 35 plants died over winter and no plants died between the first and second measurement of the second season.

**Figure 3:**
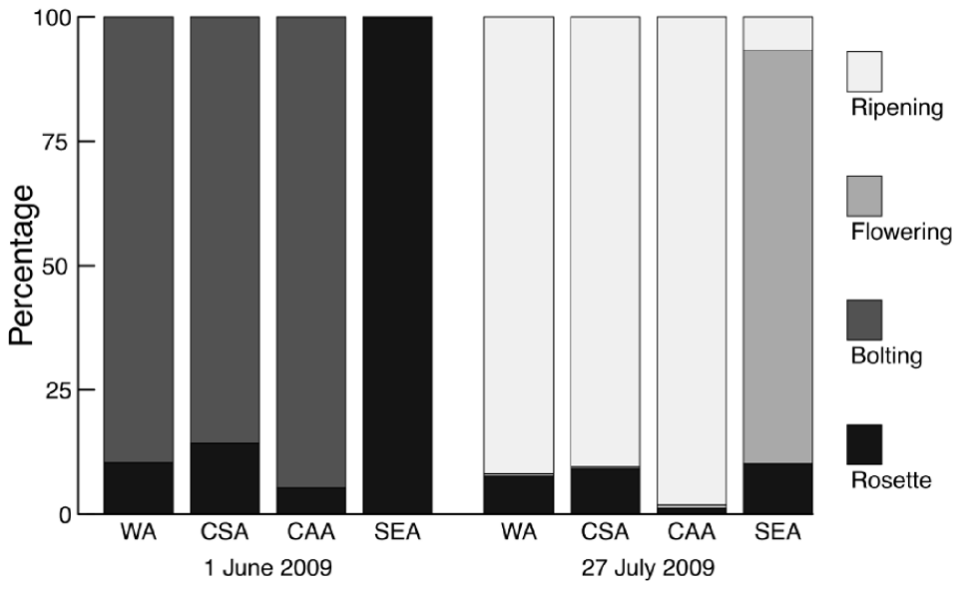
Flowering phenology of *Campanula thyrsoides* plants from four phylogeographic regions grown in a common garden. Shown is the percentage of plants in four distinct phenological stages (rosette, bolting, flowering, ripening) of surviving *C. thyrsoides* plants at two census dates. WA – Western Alps; CSA – Central Swiss Alps; CAA – Central Austrian Alps; SEA – South-eastern Alps.

By the first measurement in 2009, the majority of surviving plants of WA, CSA and CAA had already started bolting, whereas SEA plants showed no sign of initiation of flowering (Fig. 3). By the second measurement in 2009, the majority of surviving plants from WA, CSA and CAA were in the seed-ripening stage, whereas only 7% of SEA plants reached the seed-ripening stage and 83% were flowering (Fig. 3). *G* tests showed that, during both censuses, phenological states differed significantly among regions (*P*<0.0001) and populations within regions (*P*<0.01), except for SEA populations during the first census (*P* = 1) and CAA populations during the second census (*P* = 0.44). Most SEA plants had finished flowering only on 20 October 2009, when snow and frost hampered further growth (data not shown).

### Past selection and local adaptation

Estimates of *Q*_RT_ and *Q*_SR_ ranged from 0.000–0.750 and 0.030–0.269, respectively (Table 3). Number of inflorescences, maximum inflorescence height and above-ground biomass showed stronger differentiation among than within regions, whereas the opposite was true for leaf length to width ratio for 2009, with the remaining traits showing no statistically significant differences. *F*'_RT_ and *F*'_SR_ were 0.092 and 0.499, respectively, and the overall *F*'_ST_  = 0.591 was comparable with Hedrick's [36] *G*'_ST_  = 0.539. Comparing molecular with quantitative differentiation indicated that, among regions, diversifying selection (*Q*_RT_ > *F*_RT_) acted on maximum inflorescence height and above-ground biomass, whereas for leaf length to width ratio in 2009 and number of leaves, selection acted against strong differentiation (*i.e.* unifying selection, *Q*_RT_ < *F*_RT_). Selection did not need to be invoked to explain the differentiation in the remaining traits (*Q*_RT_  =  *F*_RT_). Within regions, all traits except number of flowers showed significantly lower differentiation compared to molecular differentiation (*Q*_SR_ < *F*'_SR_), indicating that selection restricted differentiation for these traits.

**Table 3:** Differentiation in phenotypic traits and molecular markers among and within phylogeographic lineages of *Campanula thyrsoides*.

Phenotypic differentiation	*Q*_RT_	*Q*_SR_	*Q*_RT_ vs. *Q*_SR_
Leaf length to width ratio 2008	0.093 (0.079–0.107) ns	0.213 (0.097–0.329) ↓	=
Leaf length to width ratio 2009	0.000 (0.000–0.000) ↓	0.202 (0.101–0.303) ↓	<
Number of leaves	0.062 (0.052–0.072) ↓	0.092 (0.015–0.170) ↓	=
Number of inflorescences	0.106 (0.097–0.116) ns	0.030 (0.022–0.038) ↓	>
Maximum inflorescence height	0.371 (0.355–0.387) ↑	0.230 (0.157–0.304) ↓	>
Number of flowers	0.097 (0.030–0.164) ns	0.269 (0.013–0.526) ns	=
Above-ground biomass	0.750 (0.698–0.802) ↑	0.121 (0.000–0.284) ↓	>
Molecular differentiation	*F*'_RT_	*F*'_SR_	
	0.092 (0.080–0.105)	0.499 (0.488–0.510)	

*Q*_RT_ – quantitative genetic differentiation (95% CI) among four phylogeographic regions; *Q*_SR_ – quantitative genetic differentiation (95% CI) among 21 populations within four regions; *F*'_RT_ – molecular differentiation (95% CI) among four phylogeographic regions based on microsatellite data; *F*'_SR_ – molecular differentiation (95% CI) among 21 populations within four regions based on microsatellite data; ↓– unifying selection in phenotypic trait; ↑– diversifying selection in phenotypic trait; ns – non-significant difference between phenotypic trait and molecular differentiation; *Q*_RT_ vs. *Q*_SR_: ns – non-significant difference between differentiation during glaciation and after postglacial recolonisation; *Q*_RT_ > *Q*_SR_ – significantly larger differentiation during glaciation than after postglacial recolonisation; *Q*_RT_ < *Q*_SR_ – significantly larger differentiation after postglacial recolonisation than during glaciation.

Correlations between trait values measured in the common garden and elevation of origin were significant for the number of inflorescences, maximum inflorescence height and above-ground biomass (Table 4). When correlating trait values of WA, CSA and CAA populations with elevation of origin, number of inflorescences and biomass decreased in strength of significance, height even became insignificant, but leaf length to width ratio in 2009 and number of flowers became significant. In the SEA populations, elevation of origin correlated significantly only with biomass.

**Table 4:** Correlations between measured plant traits in the common garden and elevation of origin in *Campanula thyrsoides* for different sets of populations.

	All regions^1^	WA, CSA, CAA^2^	SEA^3^
	*r*	*r*	*r*
Post-transplantation survival	0.13	−0.26	0.79
Leaf length to width ratio 2008	−0.38	−0.24	−0.37
Leaf length to width ratio 2009	−0.27	−0.53 *	−0.53
Number of leaves	0.26	−0.46	−0.13
Number of inflorescences	−0.70 ***	−0.64 **	−0.73
Maximum inflorescence height	−0.82 ****	−0.46	−0.94
Number of flowers	0.04	−0.50 *	−0.55
Above-ground biomass	−0.93 ****	−0.61 *	−0.97 *

1 All populations from all regions (*n* = 21); ^2^ *WA*, *CSA* and *CAA* populations (subsp. *thyrsoides*; *n* = 17); ^3^ *SEA* populations (subsp. *carniolica*; *n* = 4). *r* – Pearson's correlation coefficient. Sequential Holm-Bonferroni corrected *P*-values refer to: * *P*<0.05; ** *P*<0.01; *** *P*<0.001; **** *P*<0.0001.

## Discussion

The regional differentiation of morphological and phenological traits among phylogeographic regions within *C. thyrsoides* confirms our hypothesis that phenotypic differentiation mirrors the current molecular structure of four longitudinally oriented phylogeographic regions in the European Alps. Since this current phylogeography is likely a result of glacial survival in isolated refugia [12], the observed congruence between molecular and phenotypic patterns suggests that glacial history may have driven the evolution of both [7]. It is unlikely that regional differentiation is potentially confounded by geographic distance-related effects of genetic drift or adaptation to Alpine-wide environmental gradients, because we included the mean Euclidean geographic distance of each focal population to all other populations as a covariate in the models. The covariate effect of initial rosette diameter (which may be caused by variable germination dates, genetic variation among plants or maternal effects) was stronger in early life-traits than in reproductive traits. This suggests a diminishing influence of initial rosette size over time, as has been found in other studies (*e.g.* [38], [39]). Thus, apart from the strong effects of rosette diameter on early survival, any potential effects of initial size were not of great importance with respect to lifetime reproductive success after transplanted plants managed to establish themselves.

The observation of regional differentiation in phenotypic traits, explained as signature of the effects of glacial survival, shows that glacial history can have a long-lasting influence. Due to the current, strong isolation of *C. thyrsoides* populations in a heterogeneous environment and limited seed and insect-mediated pollen dispersal [40], [41], gene flow between populations since postglacial recolonisation is probably weak though not absent [12], [42]. This may have prevented homogenisation of phenotypic differentiation across populations from the phylogeographic regions, although considerable genetic introgression may be present between populations from the CSA and CAA regions (Fig. S1; [12]). In addition, topographical features (such as the wide Aosta valley) and edaphic factors (such as the magnesium-rich Dolomites) may obstruct dispersal between neighbouring regions, causing regional differentiation. The phenotypic patterns that we presume to be largely the result of differentiation during glacial survival in refugia may therefore in part be due to current processes.

### Regional adaptation

Regional differentiation of phenotypic traits may have evolved due to genetic drift, adaptation during survival in glacial refugia, or a combination of both. Similarly, differentiation among populations within regions can result from drift or selection after postglacial recolonisation. We presume that values of *Q*_RT_ and *Q*_SR_ represent these two temporal phases of phenotypic differentiation in the measured traits, *i.e.* during glacial survival and after subsequent postglacial recolonisation, respectively. In a similar way, *F*'_RT_ and *F*'_SR_ represent (mainly) genetic drift among regions and among populations within regions, respectively (*cf.* [22]). The amount of phenotypic and molecular differentiation during the respective periods can be compared, and *Q*_RT_–*F*'_RT_ and *Q*_SR_– *F*'_SR_ comparisons can be used to infer whether, in addition to drift, selection acted on phenotypic traits during glacial survival and after postglacial recolonisation, respectively [23].

*F*'_RT_ was much lower than *F*'_SR_, suggesting that genetic drift played a stronger role after postglacial recolonisation than during glacial survival. It is well conceivable that drift is particularly strong during recolonisation as populations were probably small and experienced numerous founder effects. Such a scenario is supported by theory and empirical data [43] and is known as ‘surfing’. Another possible explanation, in which initially strong regional differentiation in molecular markers decreases after recolonisation due to admixture among regions, was not supported by the molecular data [13].

Three important life-history traits (*i.e.* number of inflorescences, height and biomass) diverged more strongly during glacial survival than after postglacial recolonisation, and diversifying selection acted on height and biomass during glacial survival. These regional phenotypic changes most likely reflect the strong morphological and phenological differences between the two subspecies [24]. The observed delayed flowering of SEA plants (subsp. *carniolica*) is considered adaptive to the long sub-mediterranean summers and contrasts with the earlier flowering optimal for plants from the other regions (subsp. *thyrsoides*) growing at higher elevations [24]. For plants experiencing a long growing season, delayed flowering allows for prolonged build-up of reserves and therefore results in higher seed production (*i.e.* number or weight), whereas a short growing season at higher elevations (or latitudes) selects for early onset of flowering and rapid fulfilment of the life cycle [44]–[47]. Delayed flowering was also observed in natural populations of SEA, thereby confirming that the observed delay is not merely a response to the common garden environment [24].

The ultimate cause for the strong morphological and phenological differentiation of SEA populations versus WA, CSA and CAA populations could be that the south-eastern lineage of *C. thyrsoides* has survived at least the Last Glacial Maximum *in situ* under presumably relatively benign environmental conditions compared with WA, CSA and CAA lineages, which probably survived *ex situ* in refugia under presumably harsh conditions along the northern periphery of the Alps, causing differentiation through adaptation to the different climates [11], [12], [24], [26], [30]. This may have caused the current elevational distribution, with colline/montane populations from the South-eastern Alps experiencing a prolonged season compared to sub-alpine/alpine populations from the other regions. The distributional and temporal reproductive isolation between the two subspecies could eventually lead to glacial history-driven allopatric speciation [24].

Clipped plants generally suffered negative effects on traits, but the clipping×region and clipping×population interactions were exceptions to this trend, with some populations even showing overcompensation [48]. Historical data on grazing regimes in the investigated regions and populations would allow testing for a relationship with susceptibility to grazing, which would suggest adaptation to grazing regimes.

### Postglacial differentiation and adaptation

Populations within regions were differentiated in all measured traits except survival, and *Q–F*' comparisons indicated that unifying selection acted on all traits except number of flowers during recolonisation, thus restricting though not prohibiting differentiation. It is important to note that unifying selection does not necessarily imply that stasis was selected for, but that differentiation is not as strong as expected from random drift [49]; in most traits, considerable differentiation within regions likely resulted from selection restricting, but not prohibiting, phenotypic change, which led to adaptations to the spatially and temporally heterogeneous Alpine landscape. In the light of the observed strong molecular drift during recolonisation (*i.e.* surfing [43]), it is not surprising that selection restricted phenotypic differentiation as the effects of drift may well have led to maladaptions.

We included five populations from a small but heterogeneous region in CSA (Schynige Platte, 10 km^2^; Table S1). Their remarkable population differentiation in various traits indicates that even at small spatial scales, populations may be substantially differentiated, either as the result of genetic drift or as an adaptation to their local environments. However, such differentiation is only possible when gene flow is restricted, as has recently been found for populations from this particular area [40], [41] (but see [42]).

The negative correlations of elevation of origin with number of inflorescences, maximum inflorescence height and above-ground biomass measured in the common garden suggest postglacial adaptation to climatic variables related to elevation across the European Alps. Monty and Mahy [10] found similar negative relationships for final height and above-ground biomass in a common garden experiment with *Senecio inaequidens* originating from two contrasting elevational transects from northern Belgium and the French Pyrenees, which this species had colonised *c.* 1950 CE. Plant height also decreased with elevation of origin in a study on *Festuca eskia* by Gonzalo-Turpin and Hazard [50], and plant size as well as vegetative and reproductive investment decreased with elevation of origin in the sub-alpine/alpine fodder grass, *Poa alpina* [51], and in the grassland herb, *C. barbata* [8]. The phenomenon of decreasing size with increasing elevation has been explained as an adaptation to harsher conditions and shorter growing seasons [52], [53]. Therefore, this well-established growth pattern across (functionally different) species holds for the inflorescences of the monocarpic *C. thyrsoides*.

### Among- versus within-region differentiation

It does not seem that the ongoing differentiation within regions and admixture among regions over time is having an eroding effect on historical signatures in distinct traits, since regional effects were still very present. In fact, effects of glacial survival on phylogeographic differentiation are currently stronger than postglacial differentiation for important phenotypic traits. A similar result (*i.e.* higher regional compared to population differentiation for various growth, reproductive and morphological traits) was found in the alpine clonal plant, *Geum reptans* [7]. In contrast, stronger differentiation within regions compared to among regions has been found in the majority of investigated phenotypic traits in the related species, *Campanula barbata* [8]. This may be explained by *C. barbata*'s broad distribution and supposedly high genetic connectivity among populations in the Alpine landscape, eroding the glacial signature. This is in contrast to the rare and isolated occurrences of *C. thyrsoides* and *G. reptans*, which presumably hamper gene flow. However, these interspecific differences may also be related to species-specific differences in glacial history, which in turn may be due to the ecology of the species or to chance effects.

## Conclusions

We showed that regional phenotypic differentiation in the Alpine plant, *C. thyrsoides*, is in line with patterns of neutral molecular differentiation, suggesting that glacial history is jointly responsible for this differentiation and that the phylogeographic lineages diverged independently in refugia during glaciation. Our results moreover indicated that diversifying selection acted in glacial refugia, which is most clearly apparent in the strongly diverging phenology between the two subspecies. Postglacial differentiation was likewise due to selection driving adaptation to the heterogeneous landscape of the Alps. This inference was supported by negative correlation coefficients between traits and elevation of origin. We conclude that, irrespective of adaptation of plants to their current environment, glacial history can have a strong and long-lasting influence on the phenotypic evolution of widespread Alpine plants.

## Supporting Information

Figure S1 **Microsatellite marker differentiation among individuals from 51 populations of** ***Campanula thyrsoides*** **sampled from the European Alps for** ***K*** **= 4 clusters inferred from Bayesian cluster analysis using the program STRUCTURE (Pritchard et al., 2000).** Populations are aligned West-East and different colours indicate the different phylogeographic clusters (regions) indicated by their respective names. The populations sampled for the current study are indicated by their abbreviations (see Table S1 in Supporting Information). The graph shows the simulation run with the maximum likelihood for the posterior distribution, out of 20 runs. A total of 17 out of 21 of the sampled populations in the current study are part of this STRUCTURE analysis. For detailed discussion of these results, see Kuss et al., 2011 [13].
(DOC)

Table S1 **Location, geographic coordinates (WGS 84) and altitude (m a.s.l.) of 21 sampled** ***Campanula thyrsoides*** **populations across the Alps and Jura Mountains.**
(DOC)
