# Supplementary material for: Glacial History Affected Phenotypic Differentiation in the Alpine Plant, Campanula thyrsoides
Source: PLoS One. 2013 Oct 16;8(10):e73854. doi: 10.1371/journal.pone.0073854 (PMC3797877; doi:10.1371/journal.pone.0073854)
Supplement: Figure S1 — Microsatellite marker differentiation among individuals from 51 populations of Campanula thyrsoides sampled from the European Alps for K = 4 clusters inferred from Bayesian cluster analysis using the program STRUCTURE (Pritchard et al., 2000). Populations are aligned West-East and different colours indicate the different phylogeographic clusters (regions) indicated by their respective names. The populations sampled for the current study are indicated by their abbreviations (see Table S1 in Supporting Information). The graph shows the simulation run with the maximum likelihood for the posterior distribution, out of 20 runs. A total of 17 out of 21 of the sampled populations in the current study are part of this STRUCTURE analysis. For detailed discussion of these results, see Kuss et al., 2011 [13]. (DOC) [file pone.0073854.s001.doc]

**Supporting Information**

Scheepens, J. F., Frei, E. S., Stöcklin, J. 2013. Glacial history affected phenotypic differentiation in the Alpine plant, *Campanula thyrsoides*.

**Figure S1.** Microsatellite marker differentiation among individuals from 51 populations of *Campanula thyrsoides* sampled from the European Alps for *K* = 4 clusters inferred from Bayesian cluster analysis using the program STRUCTURE (Pritchard et al., 2000). Populations are aligned West-East and different colours indicate the different phylogeographic clusters (regions) indicated by their respective names. The populations sampled for the current study are indicated by their abbreviations (see Table S1 in Supporting Information). The graph shows the simulation run with the maximum likelihood for the posterior distribution, out of 20 runs. A total of 17 out of 21 of the sampled populations in the current study are part of this STRUCTURE analysis. For detailed discussion of these results, see Kuss et al., 2011 [13].

**
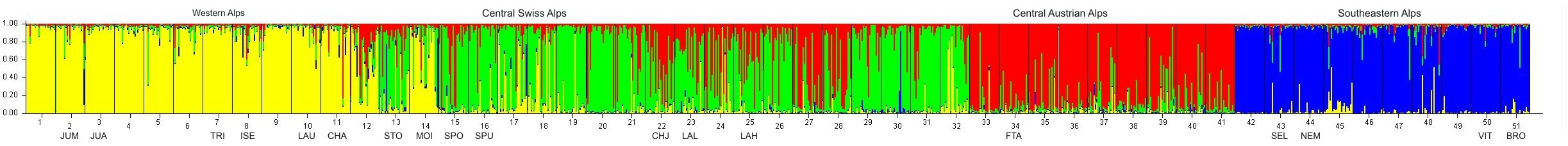
**

**References** (not in main text)

Pritchard, J. K., Stephens, M. & Donnelly, P. (2000) Inference of population structure using multilocus genotype data. Genetics 155: 945–959.
