# Supplementary material for: Glacial History Affected Phenotypic Differentiation in the Alpine Plant, Campanula thyrsoides
Source: PLoS One. 2013 Oct 16;8(10):e73854. doi: 10.1371/journal.pone.0073854 (PMC3797877; doi:10.1371/journal.pone.0073854)
Supplement: Table S1 — Location, geographic coordinates (WGS 84) and altitude (m a.s.l.) of 21 sampled Campanula thyrsoides populations across the Alps and Jura Mountains. (DOC) [file pone.0073854.s002.doc]

**Supporting Information**

Scheepens, J. F., Frei, E. S., Stöcklin, J. 2013. Glacial history affected phenotypic differentiation in the Alpine plant, *Campanula thyrsoides*.

**Table S1.** Location, geographic coordinates (WGS 84) and altitude (m a.s.l.) of 21 sampled *Campanula thyrsoides* populations across the Alps and Jura Mountains.

| Location | Code | Region | Northing | Easting | Altitude | *n* |
| --- | --- | --- | --- | --- | --- | --- |
| Jura, Les Amburnez | JUA | WA | 46°32'27.52" | 6°13'58.57" | 1340 | 48 |
| Jura, Col du Marchairuz | JUM | WA | 46°33'06.27" | 6°15'13.46" | 1440 | 47 |
| Le Chazelet | CHA | WA | 45°03'23.51" | 6°16'55.49" | 1757 | 46 |
| Col du Lautaret | LAU | WA | 45°02'03.17" | 6°23'59.63" | 2025 | 47 |
| Trient, Les Tseppes | TRI | WA | 46°02'53.93" | 6°58'47.05" | 2020 | 48 |
| Col d'Iseran | ISE | WA | 45°23'10.42" | 7°02'50.81" | 2212 | 48 |
| Lac du Moiry | MOI | WA | 46°08'12.78" | 7°34'02.87" | 2266 | 48 |
| Stockhorn | STO | CSA | 46°41'37.05" | 7°32'17.05" | 2148 | 38 |
| Schynige Platte 4 | SP4 | CSA | 46°39'17.31" | 7°54'16.67" | 1911 | 48 |
| Schynige Platte 6 | SP6 | CSA | 46°39'15.23" | 7°54'19.79" | 1916 | 48 |
| Schynige Platte 18 | SP18 | CSA | 46°39'33.73" | 7°55'14.41" | 1930 | 48 |
| Schynige Platte 22 | SP22 | CSA | 46°39'46.86" | 7°55'57.57" | 2022 | 44 |
| Schynige Platte 23 | SP23 | CSA | 46°39'46.12" | 7°56'14.21" | 1958 | 49 |
| Churwalden, Joch | CHJ | CAA | 46°47'51.41" | 9°33'53.65" | 1890 | 48 |
| Langwies, Holzbüel | LAH | CAA | 46°49'41.97" | 9°44'00.53" | 1700 | 42 |
| Langwies, Listboden | LAL | CAA | 46°51'07.02" | 9°45'32.22" | 2000 | 48 |
| Ftan, Prui | FTA | CAA | 46°48'32.68" | 10°13'20.37" | 2101 | 49 |
| Sella Nevea | SEL | SEA | 46°23'35.00" | 13°27'46.00" | 932 | 28 |
| Nemski Rovt | NEM | SEA | 46°16'23.50" | 13°58'30.00" | 663 | 49 |
| Brodnice | BRO | SEA | 46°06'24.30" | 15°16'53.10" | 283 | 42 |
| Vitanje | VIT | SEA | 46°22'27.80" | 15°17'16.90" | 422 | 40 |

Code – abbreviation as used in the main text, Region – Phylogeographic region: *WA* Western Alps (including Jura Mts.), *CSA* Central Swiss Alps, *CAA* Central Austrian Alps, *SEA* South-eastern Alps, *n* – sample size of individuals used in the common garden. Regions according to Kuss et al. 2011 [13].
